# Study of Active Ingredients in Black Soybean Sprouts and Their Safety in Cosmetic Use

**DOI:** 10.3390/molecules171011669

**Published:** 2012-10-01

**Authors:** Jixiang Lai, Can Xin, Ya Zhao, Bing Feng, Congfen He, Yinmao Dong, Yun Fang, Shaomin Wei

**Affiliations:** 1 School of Chemical and Material Engineering, Jiangnan University, Wuxi 214122, Jiangsu, China; 2 School of Chemical Engineering, Hebei United University, Tangshan 063009, Hebei, China; 3 Shanghai Jahwa United Co. Ltd, Shanghai 200082, China; 4 School of Science, Beijing Technology and Business University, Beijing 100048, China

**Keywords:** additives, cosmetics, black soybean sprouts, anti-oxidation, whitening, safety

## Abstract

Active ingredients in different lengths of black soybean sprouts were extracted with water. Concentrations of the main proteins and polysaccharides were determined by the Forint phenol assay and phenol-sulfuric acid assay, respectively. Anti-oxidizing capacities of the extracts were measured *in vitro* using the DPPH scavenging test and whitening capacity was measured *in vitro* using the tyrosinase inhibition test. The effects of the bean sprout extracts on human skin fibroblasts damnified by H_2_O_2_ were studied using an MTT colorimetric assay. The safety of the extracts was determined using the red blood cell (RBC) test, chick chorioallantoic membrane (CAM) assay and human patch test. Results show that DPPH radical scavenging rates at different shoot lengths were all greater than 95%, while the tyrosinase inhibition capacity of the extracts reached 98%. Hemolysis rate in all extracts were lower than 10%, below the 20% regulatory limit for the RBC test. No signs of allergic reactions were observed in the human patch tests. The optimum extract was obtained from bean sprouts grown to 0.5 cm. Extracts of black bean sprouts are safe and can be used as additives in anti-aging and whitening cosmetic products.

## 1. Introduction

Black soybean (*Glycine max* var) is the black seed of the soybean *Glycine max* (L.) merr, also known as the black bean. The *Supplement to Compendium of Materia Medica* states that black beans can be beneficial to sperm and bone marrow production, muscle strength, hair growth, and the immune system. Modern scientific research shows that black beans have hypolipidemic and antioxidant properties and can be used to beautify the skin [1]. 

Studies have shown that more protein can be extracted from the bud of germinated black beans. The proteins, polysaccharides and mineral elements are released by enzyme activity, resulting in greater absorption and uptake by the body [2]. Black bean sprouts are rich in calcium, phosphorus, iron, potassium and vitamins, where the level of vitamins increases in the budding process [3]. This makes the extraction easier: germination is a natural way to extract the active ingredient in the seeds, and the ingredients are easy absorbed by the body [4]. However, most studies of black bean sprouts have mainly concentrated on their material composition and development as a functional food. Little research has been devoted to the use of black bean sprouts in beauty products.

## 2. Results and Discussion

### 2.1. Concentrations of Proteins and Polysaccharides in Extracts

**Figure 1:**
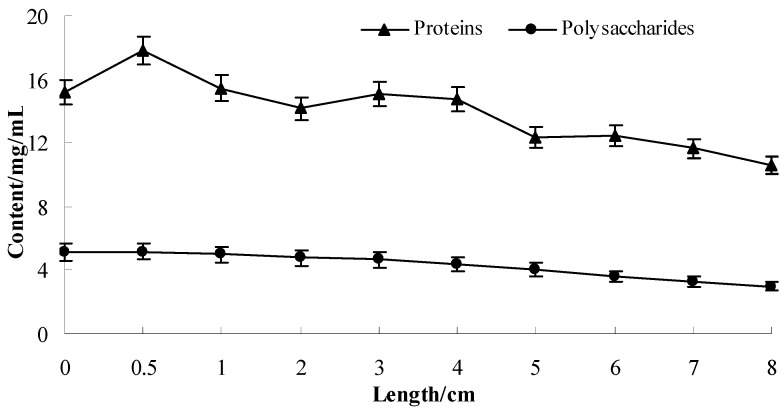
Concentration (mg/mL) of proteins and polysaccharides in soybean sprout extracts taken from different lengths of bean sprouts (n = 3).

The concentrations of proteins and polysaccharides in water extracts are shown in Figure 1. Each sample was measured three times and the mean value recorded as a detection result.

The level of protein was found to change over the course of the germination process, peaking when the sprout measured 0.5 cm and reaching a highest protein content of 18 mg/mL. The analysis of variance showed that there was significant difference in the protein content between 0.5 cm sprout and black soybean seeds (*p* < 0.05). This trend is consistent with its cosmetic effects. Protein concentrations gradually declined with further growth of the sprout. According to Hyun *et al.* [5], proteins stored in black bean seeds (mainly glycinin and β-conglycinin) degrade during germination, concurrent with a slight increase in protein content in the 0.5 cm sprout extract. Subsequently, proteins are consumed gradually during germination. Polysaccharide content declined from more than 5 mg/mL in germinating seeds (0.5 cm) to 3.5 mg/mL in sprouts (10 cm); this decline may be attributed to sugar consumption to provide energy [6,7]. However, the components which play a major role in the antioxidant and skin-whitening effects of black soybean remain to be elucidated.

### 2.2. Cosmetic Activity

#### 2.2.1. Antioxidant Activity

**Figure 2:**
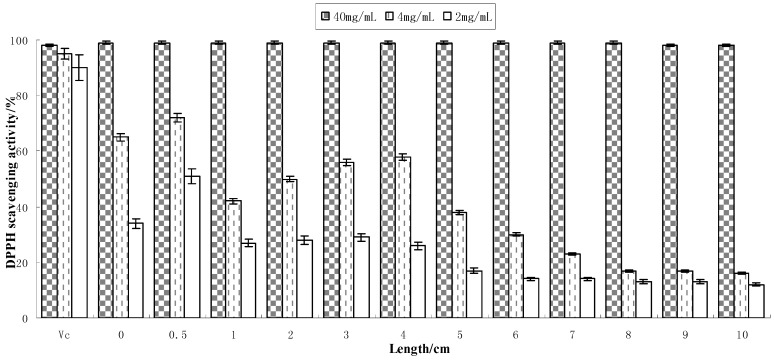
Anti-oxidizing capacity of extracts obtained from different lengths of bean sprouts (n = 3).

The anti-oxidizing capacities of black soybean sprout extracts (total soluble solid content: 40, 4 and 2 mg/mL) at different lengths (0–10 cm) are shown in Figure 2.

DPPH radical scavenging rates in the extracts (40 mg/mL) at different shoot lengths were all greater than 95%, similar to the activity of vitamin C (commonly added as 1%–2% in cosmetics). Scavenging rates reached more than 70% after 10-fold dilution (4 mg/mL) and about 50% after 20-fold dilution (2 mg/mL). At these two concentrations, analysis of variance showed significant differences in the antioxidant activity among sprouts of 12 lengths; regression analyses (R^2^ = 0.86) between different length sprouts and antioxidant activity was significant, indicating that bud length did influence the antioxidant activity. Extracts of sprouts measuring 0.5 cm in length had the highest scavenging rate at various concentrations, possibly due to the budding process which increases levels of smaller components and polypeptides which strengthen the anti-oxidizing capacity. Fernandez-Orozco *et al.* [8] found that the budding process can increase the antioxidant activity of soybean extracts and our results are consistent with this study. The findings of Zielinski [9] also agree with our results. Soybean proteins have higher oxidation activity following enzymatic hydrolysis than dry seeds according to the study by Liu *et al.* [10], due to the greater free radical scavenging efficacy of peptides generated by enzymatic hydrolysis. Vitamin C is a commonly used antioxidant with stronger activity but less stability (vitamin C may be inactivated by high temperatures, alkalinity or long periods of illumination). Soybean sprout extracts (40 mg/mL) appear to have considerable antioxidant activity, similar to that of vitamin C, and greater stability (resisting high and low temperatures, pH 6.0–10.0 and long periods of illumination), so have good potential for use in food and cosmetic products as a new antioxidant.

#### 2.2.2. Whitening Capacity

**Figure 3:**
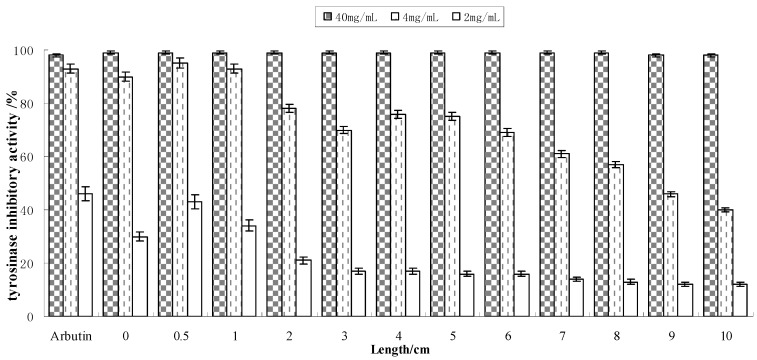
Tyrosinase-inhibition capacity of extracts obtained from different lengths of bean sprouts (n = 3).

Tyrosinase is a rate-limiting enzyme in melanin synthesis. Inhibition of tyrosinase can reduce the produce of melanin to get a whitening effect, so the stronger an active ingredient is in inhibiting tyrosinase, the greater whitening effect it has. Tyrosinase-inhibiting capacities of the black bean extracts (total soluble solid content: 40, 4 and 2 mg/mL) at different sprout lengths (0–10 cm) are shown in Figure 3.

Tyrosinase inhibition capacity of the extracts (40 mg/mL) at different shoot lengths reached 98%, which is better than the activity of arbutin (normally added as 1%–5% in cosmetics). Inhibition capacity reached 60%–95% after 10-fold dilution (4 mg/mL) and about 40% after 20-fold dilution (2 mg/mL). In these two concentrations, analysis of variance showed significant differences in the tyrosinase inhibition activity among sprouts at 12 lengths; regression analyses (R^2^ = 0.62) between different length sprouts and tyrosinase inhibition activity was significant, indicating that bud length did influence the whitening capacity. Thus, soybean extracts have strong inhibition capacity and can be used as a whitening ingredient in cosmetics. Extracts from sprouts grown to 0.5 cm had the highest capacity in various concentrations.

#### 2.2.3. Effect on Anti-H_2_O_2_-Induced Damaged Skin Fibroblast

**Figure 4:**
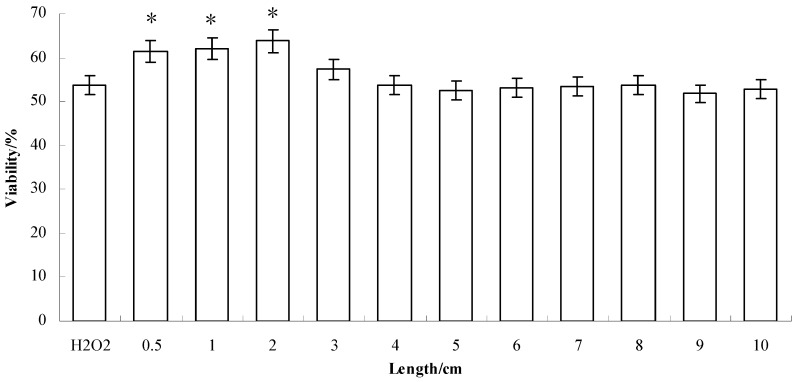
Effects of extracts of bean sprouts grown to different lengths on human skin fibroblasts damnified by H_2_O_2_. (n = 3) (* *p* < 0.05).

The effects of the black soybean extracts (40 mg/mL) on anti-H_2_O_2_-induced damaged skin fibroblasts (taken from sprouts of 0.5–10 cm) were measured by the MTT assay. We analysed our results using a similar method to that used by Xiao *et al.* [11] and found the following: when soybean sprout extracts (40 mg/mL) were first incubated with fiber cells for 3 to 12 h and then incubated with H_2_O_2_ (800 μmol/L) for 4 h, cell viability did not increase significantly. However, when samples were subjected to early incubation for up to 24 h and then incubated with H_2_O_2_ (800 μmol/L) for 4 h, the extracts (40 mg/mL) showed significant resistance (*p* < 0.05) to H_2_O_2_-induced cell damage in comparison with blank control (the H_2_O_2_ group). The activity of the extracts increased to 61.5 ± 5.26%, 61.9 ± 4.11% and 63.8 ± 4.87% for bean sprout lengths of 0.5, 1 and 2 cm length respectively (See in Figure 4). Thus, the effect of the soybean extracts on H_2_O_2_-induced cell injury does not involve a chemical reaction or direct free radical scavenging; this mechanism needs further elucidation.

Antioxidant activity relates to the anti-aging effect and reflects the effectiveness of anti-aging on the human body. Tyrosinase inhibiting activity relates to the whitening effect and reflects the strength of its whitening effect. Effect on anti-H_2_O_2_-induced damaged skin fibroblast reflects the anti-aging effects on the human skin indirectly. In summary, evaluations show that soybean sprout extracts have strong cosmetic activity and can be used as anti-aging or whitening ingredient in cosmetics.

### 2.3. Safety

#### 2.3.1. Results of RBC Test

**Figure 5:**
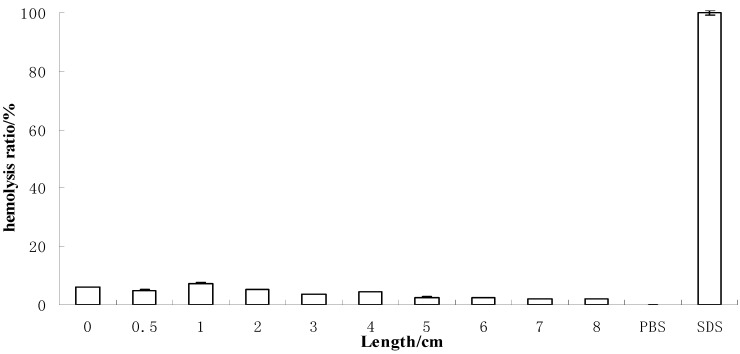
RBC test results for extracts (40 mg/mL) obtained from different lengths of bean sprouts (n = 3).

A sample can be deemed to be nonirritating if its RBC test results are lower than 20%, according to current regulations. Our RBC test results are shown in Figure 5, where the rates of hemolysis of soybean sprout extracts were all lower than 10%. 

#### 2.3.2. Results of CAM Assay

**Figure 6:**
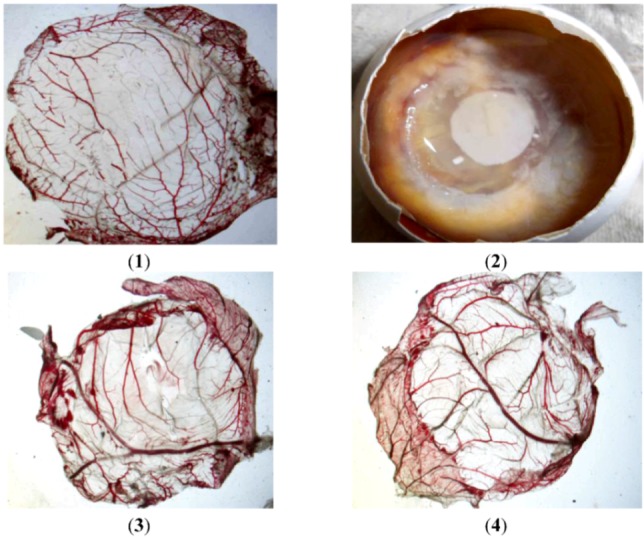
Photographs from CAM assay taken during vascular morphological observation of chick embryos: (**1**) negative control (0.9% normal saline); (**2**) positive control (0.4% SDS); (**3**) soybean sprout extract (40 mg/mL); (**4**) soybean sprout extract (4 mg/mL).

The CAM results are shown in Table 1, where the chick embryos of the positive control groups all died after 72 h while the sample groups all survived. Photographs of the CAM model taken during vascular morphological observation are shown in Figure 6 (1)–(4). Statistical analysis using software SPSS 19.0 showed that there was no significant difference between sample groups and negative control group (*p* > 0.05), but highly significant difference between sample groups and positive control groups (*p* < 0.01). The results show that the effect of sample groups on CAM was similar to normal saline (negative control) without irritation.

**Table 1:** Results of the morphological observation and the survival rate of chick embryos in CAM assay.

Group	Change of capillary ending	Change of capillary network	Vascular change	Change of CAM and the protein	Irritation	Survival rate after 72 h/%
Negative control	no hyperemia, no exudation	clear morphology, no visual change	clear morphological structure and profile, no change	no visual change	No	100
Positive control (4%SDS)	obvious hyperemia, exudation	obvious fade, hemoglobin degeneration, only profile left	hyperemia, morphological structure change	protein denaturation,color change	Serious	0
Positive control (0.4%SDS)	obvious hyperemia, exudation	obvious fade, hemoglobin degeneration, only profile left	hyperemia, morphological structure change	protein denaturation,color change	Serious	0
Soybean (40 mg/mL)	slight hyperemia, no exudation	clear morphology, no visual change	clear morphological structure and profile, no change	no visual change	No	100
Soybean (4 mg/mL)	slight hyperemia, no exudation	clear morphology, no visual change	clear morphological structure and profile, no change	no visual change	No	100

#### 2.3.3. Human Skin Patch Test Results

The results of the human skin patch test showed that thirty people who took part in the study all showed a Grade 0 response; no signs of allergic reactions were observed in the test, meaning that the bean sprout extracts did not cause any allergic reactions on human skin. 

Safety assays play a very important role in the safety evaluation of cosmetic materials with the characteristics of simplicity, time-savings and low-cost, but they still cannot fully replace human skin tests because of its specialized function and too simple evaluation index. In conclusion, all safety evaluation results above indicate that the black soybean sprout extracts are safe to use in products intended for application on human skin.

## 3. Experimental

### 3.1. Reagents and Materials

The following reagents and materials were obtained for this study: soybeans (*Glycine max* var) (Shijiazhuang, China); 1,1-diphenyl-2-picrylhydrazyl radical (DPPH•) and tyrosinase, AR (Biodee Biotechnology Co. Ltd, Beijing, China); arbutin and vitamin C, AR (Beijing Chemical Reagent Company, Beijing, China); DMEM medium and fetal bovine serum (Gibco, New York, NY, USA); H_2_O_2_ (3%), MTT and dimethyl sulfoxide (DMSO) (Sigma-Aldrich Corporation, St. Louis, MO, USA).

### 3.2. Extraction and Analysis of Composition

Black bean sprouts were extracted according to the method by Li *et al.* [12]. To summarize, the black beans were soaked in deionized water, germinated, harvested, dried, crushed, extracted by ultrasonification, filtered, and centrifuged to separate the components and collect the supernatant.

The mass concentrations of proteins in water extracts of black soybean sprouts were measured by the Flint-phenol method [13]. Testing solution (1.0 mL) was mixed with Folin-phenol reagent A (5.0 mL) and held at 40 °C for 10 min. Then Folin-phenol reagent B (0.5 mL) was added immediately and mixed quickly. After keeping at 40 °C for 30 min, the solution was placed in a 10.0 mL volumetric flask and made up to the mark with distilled water and shaken. Absorbance was measured at a wavelength of 630 nm.

The mass concentrations of polysaccharides in water extracts of black soybean sprouts were measured by the phenol-sulfuric acid method [14]. 5% Phenol solution (1.0 mL) was added to testing solution (2.0 mL) and mixed. After five minutes, concentrated sulfuric acid (5.0 mL) was quickly added dropwise, then the mixed solution was heated in boiling water for 60 min. After standing at ambient temperature for 15 min and cooling in cold water for 30 min, the solution absorbance was measured at a wavelength of 490 nm. 

### 3.3. Determination of Experimental Parameters

1,1-Diphenyl-2-picrylhydrazyl radical (DPPH•) is a stable organic free radical which can be used to evaluate the antioxidant capacity of samples by spectrophotometry [15]. The antioxidant activity was measured by the degree of scavenging DPPH radical at 517 nm. The antioxidant activity of water extracts (40, 4 and 2 mg/mL) was measured with vitamin C (VC) as the positive control. 

Tyrosinase is the rate-limiting enzyme in the synthesis of melanin. Inhibition of tyrosinase produces a whitening effect. The rate of tyrosinase inhibition in samples was measured at 475 nm using a UV spectrophotometer [15]. The whitening activity of the water extracts (40, 4 and 2 mg/mL) was measured with arbutin as a positive control.

The effects on anti-H_2_O_2_-induced damaged skin fibroblasts of black bean extracts (40 mg/mL) obtained from the sprouts grown to different lengths (0.5 to 10 cm, top to tail) were detected by an MTT assay according to Ren *et al.* [16] The cell viability was measured at 570 nm using a UV spectrophotometer. Third generation human skin fibroblasts were used in the experiment.

In above experiments, the color interference of the testing sample in spectrophotometric determinations can be eliminated by diluting the test substance and deducting the background value of the test sample. Each sample was measured three times and the mean value recorded as a detection result.

### 3.4. Safety Determination

Red blood cell tests, chicken chorioallantoic membrane assays and human skin patch tests were used to evaluate the safety of the black soybean extracts.

#### 3.4.1. Red Blood Cell Test (RBC)

Potential irritation by black soybean sprout extracts was detected using the red blood cell test to a final concentration of 40 mg/mL according to the method of Xue *et al.* [17,18,19]. The test employed 0.4% SDS as the positive control and PBS as the negative control. Hemolysis ratio was given in equation: H (%) = 100% × (OD_530nm (sample)_ − OD_530nm (negative control)_)/(OD_530nm (positive control)_ − OD_530nm (negative control)_). The color interference of the testing sample in RBC test can be eliminated by deducting the background value of the test sample. The sample is normally deemed to be non-irritating if the rate is below 20%.

#### 3.4.2. Chicken Chorioallantoic Membrane (CAM) Assay

Potential irritation by black bean extracts was detected using the chicken chorioallantoic membrane assay according to the methods of Wang *et al.* [20] and Bi *et al.* [21] using 0.4% and 4% SDS as positive controls and 0.9% normal saline as the negative control. Morphological changes of capillary ending, capillary network, vascular, CAM, protein and the survival rate of CAM were observed and recorded. Data were subjected to analysis using software SPSS 19.0. 

#### 3.4.3. Human Skin Patch Test

The skin toxicity of black bean sprout extracts (40 and 4 mg/mL) was detected with the human skin patch test according to the method of Zheng *et al.* [22]. Thirty volunteers (15 females and 15 males, 22 to 32 years old) were chosen; extracts were applied on their arms for 24 h. Then results were classified in 0, 1, 2, 3, 4 five grades (Grade 0: negative reaction; Grade 1: suspicious reaction, slight erythema; Grade 2: slight positive reaction, erythema; Grade 3: positive reaction, herpes; Grade 4: serious positive reaction, confluent herpes) according to the procedure set out in *Hygienic Standard for Cosmetics* [23]. A sample can be judged having adverse reactions to human skin if the number of Grade 1 responses is greater than five, or the number of Grade 2 response is greater than two, or the number of Grade 3 and higher grade response is greater than one. Otherwise, this sample can be judged safe to the human body.

### 3.5. Statistical Methods

The analyses of the data were done using the (IBM SPSS Statistics) v19.0 statistical package (IBM Corporation, New York, NY, USA). The experimental data were subjected to χ ^2^ tests, *p* < 0.05 as a significant difference. 

## 4. Conclusions

Black bean extracts have good antioxidant and tyrosinase-inhibitor properties, particularly in the bud rather than the dry seeds, where the budding process can improve the antioxidant and whitening properties of black soybean. Safety tests show black bean extracts to be safe and nonirritating to human skin, and thus show potential as an additive for cosmetic products.

## References

[1] Xu Y., Xu P., Wang X. (2009). Studies on extraction technology and stability of black soybean polysaccharides. Food Res. Dev..

[2] Huang G. (2005). Effect of germination on nutritional properties of seeds. Food Nutr..

[3] Xu M., Dong J., Zhu M. (2005). Effects of germination conditions on ascorbic acid level and yield of soybeans sprouts. J. Sci. Food Agric..

[4] Vidal-Valverde C., Frias J., Sierra I., Blazquez I., Lambein F., Kuo Y. (2002). New functional legume foods by germination: Effect on the nutritive value of beans, lentils and peas. Eur. Food Res. Technol..

[5] Kim H.T., Choi U.K., Ryu H.S., Lee S.J., Kwon O.S. (2011). Mobilization of storage proteins in soybean seed (*Glycine max* L.) during germination and seedling growth. Biochim. Biophys. Acta.

[6] Lin P., Lai H. (2006). Bioactive compounds in legumes and their germinated products. J. Agric. Food Chem..

[7] Plaza L., De Ancos B., Cano P. (2003). Nutritional and health-related compounds in sprouts and seeds of soybean (*Glycine max*), wheat (*Triticum aestivum* L.) and alfalfa (*Medicago sativa*) treated by a new drying method. Eur. Food Res. Technol..

[8] Fernandez R., Frias J., Zielinski H., Piskula M., Kozlowska H., Concepción V. (2008). Kinetic study of the antioxidant compounds and antioxidant capacity during germination of *Vigna radiata* cv. emmerald, *Glycine max* cv. Jutro and *Glycine max* cv. Merit. Food Chem..

[9] Zielinski H. (2003). Contribution of low molecular weight antioxidants to the antioxidant screen of germinated soybean seeds. Plan. Foods Hum. Nutr..

[10] Liu E., He J., Chen Z., Liu H., Li Y. (2009). Antioxidation Activity *in vitro* of Enzymic Hydrolysates from Black Soybean. J. Chin. Cereals Oils Assoc..

[11] Xiao J., Li X., Guo J. (2005). Dual action on hydrogen peroxide induced oxidative vascular endothelial cell damage by curcumin. Chin. J. Clin. Pharmacol. Ther..

[12] Li D., Song J., Liu C. (2009). Optimization of technology for ultrasonic-assisted extraction of pigments from black soybean hulls. T. Chin. Soc. Agric. Eng..

[13] Huang B., Zhang S., Tie X. (2004). Determination of proteins in Binchashuan by Forint-phenol reagent method. Zhejiang Yu Fang Yi Xue.

[14] Hui Q. (2011). Determination of Total Polysaccharides in Corn Stigma Health Beverage by Phenol-sulfuric Acid Method. Acad. Period. Farm Prod. Process..

[15] Zhao J., Lai J., He C. (2009). Extraction technology of active ingredients in plant Rhodiola and its function in cosmetics. China Surf. Deterg. Cosmet..

[16] Ren D., Du G., Zhang J. (2003). Inhibitory effect of salvianolic acids on endothelial cells damage induced by hydrogen peroxide. Chin. J. Pharm. Toxicol..

[17] Xue J., Yang X., Yang Y. (2010). Preliminary study on eye irritation with RBC hemolysis test to replace Draize test for pesticides. China Occup. Med..

[18] Zhang W., Yang X., Yang Y., Li X., Xiong X., Xie X., Tan X. (2010). Preliminary study on red blood cell haemolysis assay as an alternative method for eye irritation test. Chin. Prev. Med..

[19] Lagarto A., Vega R., Vega Y., Guerra I., Gonzalez R. (2006). Comparative study of red blood cell method in rat and calves blood as alternatives of Draize eye irritation test. Toxicol. In Vitro.

[20] Wang F., Ning L. (1999). The Application of Het Cam Test for Evaluation the Irritation of the Surfactants and Shampoos. China Surf. Deterg. Cosmet..

[21] Bi W., Shen S., Li Q. (2011). Screeing Active Fraction of Yunnan Baiyao for Inhibition of Angiogenesis. Chin. J. Exp. Tradit. Med. Formulae.

[22] Zheng Y., Xing G., Wang J., Wang S. (2008). Study on Seabuckthorn Seed Oil of Cosmetics Safety Test on Base of Supercritical CO_2_ Extraction Technology. Glob. Seabuckthorn Res. Dev..

[23] Ministry of Health of the People’s Republic of China. (2002). Hygienic Standard for Cosmetics.

